# Clinical and Histological Differences between Guided Tissue Regeneration with Acellular Dermal Matrix of Porcine Origin and Autologous Connective Tissue: An Animal Study

**DOI:** 10.3390/ma14020272

**Published:** 2021-01-07

**Authors:** Javier Aragoneses, Ana Suárez, Cinthia Rodríguez, Juan Manuel Aragoneses

**Affiliations:** 1 Department of Medicine and Medical Specialties, Faculty of Health Sciences, Universidad Alcalá de Henares, 28871 Madrid, Spain; javias511@gmail.com; 2 Department of Preclinical Dentistry, School of Biomedical Sciences, Universidad Europea de Madrid, 28670 Madrid, Spain; 3 Department of Dentistry, Universidad Federico Henriquez y Carvajal, Santo Domingo 10106, Dominican Republic; cinthiagarabitos@gmail.com; 4 Dean of the Faculty of Dentistry, Universidad Alfonso X El Sabio, 28691 Madrid, Spain; jmaragoneses@gmail.com

**Keywords:** autologous connective tissue graft, acellular dermal matrix, grafts, keratinized mucosa

## Abstract

This research aims to evaluate the clinical and histological parametric differences concerning keratinized tissue that result from two regeneration techniques, the subepithelial autologous connective tissue graft (ACTG) and the acellular dermal matrix (MD) of porcine origin, performed on surgical beds on edentulous spaces in an animal model. The parameters of the MD and ACTG groups were compared with samples of the control group (CG) after 15, 45, and 90 days. Nine female white pigs (*Sus scrofa domestica*) were used, and each animal provided 20 study areas (12 MD and 8 ACTG). At 15 days, the keratin layer thickness in the MD group was greater than those of the ACTG (25.27 vs. 19.95 μm) and the CG (21.2 μm). After 45 days, the MD and ACTG thickness values decreased but were higher than the CG. At 90 days, MD (19.46 μm) obtained a value close to that of CG, and the ACTG decreased to CG (15.53 μm, *p* < 0.001). The use of an MD may be a viable alternative to the ACTG because of its ability to provide increased keratinized tissue in comparison to the ACTG.

## 1. Introduction

In order to meet patients’ aesthetic demands, regenerative treatments that aim to increase keratinization of gums are increasingly used in the field of periodontal plastic surgery [1,2]. For years, surgical techniques using subepithelial autogenous connective tissue grafts (ACTG) have been considered the “gold standard” procedure [3,4,5,6]. However, ACTG regeneration techniques have several disadvantages, such as increased postoperative patient morbidity and a higher risk of complications, including postoperative pain, bleeding and necrosis [7,8,9], as well as aesthetic limitations [10,11,12,13,14].

Techniques that use stem cells to reconstruct oral soft tissue show promising results [15,16,17,18,19]; however, they are financially costly and require more documented cases for clinical application [8]. At present, probable alternatives are regenerative techniques that use acellular matrices. The first acellular matrix used in dentistry was derived from human cadaver skin [20,21]. Recently, other techniques involving the use of various xenogeneic matrices have demonstrated safety, viability, and efficacy [22,23,24]. In this field, porcine acellular dermal collagen matrices are an option to be considered [25]; however, the number of studies that scientifically support their use in clinical practice, and that compare histological results, are few [26]. Therefore, the purpose of this study was to evaluate variations at clinical and histological levels in the keratinized tissue in surgical wounds after the use of regenerative techniques, including acellular dermal matrix of porcine origin (MD) compared to results achieved using ACTG.

## 2. Materials and Methods 

### 2.1. Animals and Ethics

The animal research project protocol was approved by the Ethics and Animal Welfare Committee of the Puerta de Hierro University Hospital, Majadahonda, Spain. The procedures were carried out following the European (Directive 2010.63.EU) and Spanish (RD 53/2013) regulations regarding the handling and care of experimental animals. Nine healthy female domestic pigs (*Sus scrofa domestica*) were used [27]. During the course of this study, the same soft diet was implemented for all animals.

### 2.2. Surgical Procedures

Each animal provided 20 areas of study: 12 corresponded to MD, and the other 8 areas were treated by ACTG. The study was evaluated at three times: 15 days, 45 days, and 90 days after the intervention. The samples that were removed to create the surgical beds were considered as a control group (CG) of untreated tissue. The animals were premedicated intramuscularly with a combination of medetomidine hydrochloride 0.01 mg/kg (Medeson^®^, Uranus Vet, Barcelona, Spain), ketamine 5 mg/kg (Ketalar^®^, Pfizer, New York, NY, USA), midazolam 0.2 mg/kg (Combino Pharm, Barcelona, Spain), and atropine 0.02 mg/kg (Braun Medical, Oss, The Netherlands). To induce general anesthesia and to be able to perform endotracheal intubation, an intravenous injection of propofol 1 mg/kg (Fresenius Kabi, Bad Homburg, Germany) was used.

**Figure 1:**
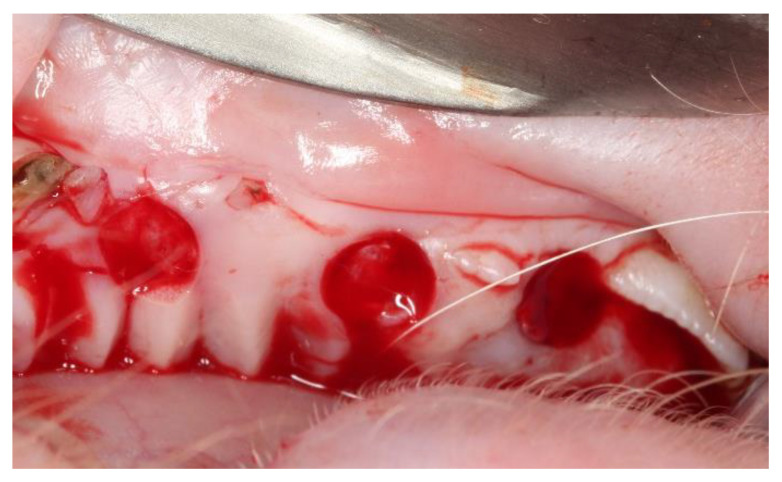
Detail of the surgical beds in an upper quadrant.

The entire surgical protocol was carried out by a single operator who proceeded to locally anesthetize with an infiltrative injection of 1.8 mL articaine with epinephrine 1/200.000 (Ultracain^®^, Hoechst, Frankfurt, Germany). Later, 5 mm diameter beds on the keratinized tissue were created with a surgical punch (5 per quadrant) of the edentulous spaces, eliminating its epithelium and leaving a layer of underlying connective tissue exposed. Out of the five surgical beds created per quadrant, three were used to place the acellular dermal matrix of porcine origin (OsteoBiol Derma^®^, Tecnoss, Giaveno, Italy), and two were used for the ACTG. Distribution was done in a randomized way (Figure 1).

**Figure 2:**
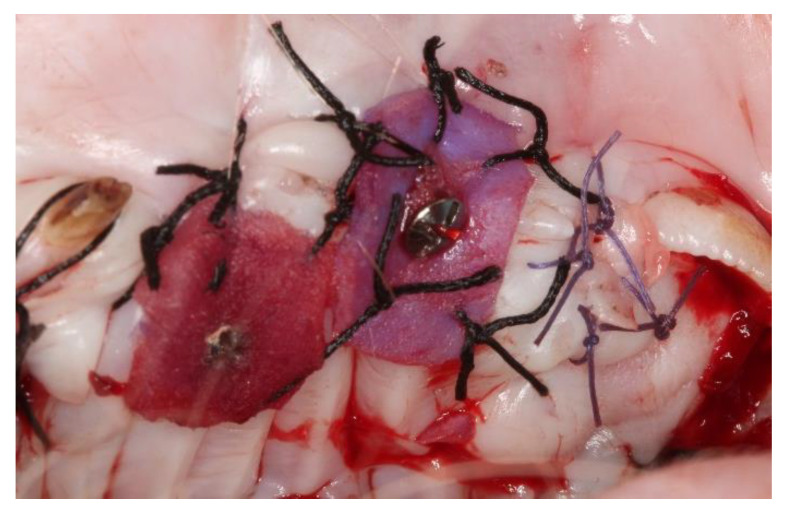
Acellular dermal matrix (center) and subepithelial autologous connective tissue graft (left) placed in surgical beds.

Placement of the MD took place without further crosslinking and was fixed by the interposition in the center of the bed of a 2 mm diameter micro-screw (Sweden & Martina, Due Carrare, Italy). Afterward, a crossed horizontal external suture was performed with a braided 5-0 single-thread silk suture and cutting needle (Sweden & Martina^®^, Due Carrare, Italy) held with a Castroviejo needle holder and 41 plain Adson Tissue pliers (Hu-Friedy Mfg. Co., Frankfurt, Germany). To obtain the autogenous connective tissue grafts from the animals’ palate, half of the palate of the selected quadrant was anesthetized with Articaine epinephrine 1/200,000 (Ultracain^®^, Hoechst, Frankfurt, Germany) using a double incision technique with a number 15C scalpel blade and a number 5 straight round scalpel handle (Hu-Friedy Mfg. Co., Frankfurt, Germany). The palate donor bed was sutured with two horizontal mattress stitches and complementary single stitches with a braided 5-0 single-thread silk suture and cutting needle (Sweden & Martina^®^, Due Carrare, Italy). The graft was divided into eight pieces of the same dimension and placed in each recipient bed with a braided 5-0 single-thread silk suture and cutting needle (Sweden & Martina^®^), sutured with a crossed horizontal external suture and fixed by the interposition in the center of the bed of a 2 mm diameter micro-screw (Sweden & Martina, Due Carrare, Italy; Figure 2).

### 2.3. Euthanasia

After obtaining the programmed biopsies, controlled and regulated slaughter of the experimental animals was carried out by a veterinarian employing an overdose of intravenous sodium pentobarbital (Pfizer, New York, NY, USA). Animals were previously sedated with medetomidine hydrochloride 0.01 mg/kg (Medeson^®^, Uranus Vet, Barcelona, Spain) and ketamine 5 mg/kg (Ketalar^®^, Pfizer, New York, NY, USA).

### 2.4. Histological Evaluation

The thicknesses of the keratin layer and the epithelium (length of epithelial ridges) were evaluated. For this purpose, all of the samples obtained at the different times studied were fixed by immersing them in a formaldehyde-neutral buffer solution of 3.7% (Sigma-Aldrich, San Luis, MO, USA) for 48 h, after which the samples were dehydrated through consecutive immersions in ethyl alcohol of 70°, 90°, and 100° (MilliporeSigma, Burlington, MA, USA). The samples were then soaked in kerosene (MilliporeSigma, Burlington, MA, USA) during the inclusion phase before being cut with a microtome to an average thickness for each section of approximately 5µm (Leica Biosystems, Nussloch, Germany). The samples were then stained with hematoxylin and eosin (MilliporeSigma, USA) for evaluation. The histomorphometric analysis was performed using a clear field optical microscope (Olympus BX41, Olympus Corporation, Shinjuku, Japan) with associated image analysis software (Image Pro Plus 6.0, Stemmer Imaging AG, Puchhem, Germany).

For each histological cut, two parameters were evaluated:

- the thickness of the keratin layer, and
- the thickness of the epithelial tissue, measured from the most coronal part of the granular layer to the more apical part of the epithelial crest.

For each parameter, three measurements of different areas were taken, and the mean of the three measurements was considered that for the histological cut. The mean of all of the histological cuts was considered the mean for every follow-up point.

### 2.5. Clinical Evaluation

Clinical evaluations were carried out by direct visualization of the animals’ oral mucosa. For this purpose, photographs were taken with a digital reflex camera (Canon EOS 650, Canon, Tokyo, Japan) with a 100 mm macro (Canon EF 100 mm f/2.8 L Macro, Canon, Tokyo, Japan) and ring flash (Macro Ring Lite MR-14EX II, Canon, Tokyo, Japan). The color and the aesthetic results obtained with both treatments were evaluated by visual assessment of the treated areas concerning the adjacent tissues of healthy keratinized gingiva. The external evaluator was unaware of the treatment carried out for each case.

### 2.6. Statistical Analysis

The mean and standard deviation were calculated for each histological parameter measured for both the experimental and the control groups. Because the groups did not follow a normal distribution of their randomized data (*p*-value < 0.005), the non-parametric Mann-Whitney U test was used for comparisons. The significance level chosen in all statistical tests was *p* < 0.05.

## 3. Results

### 3.1. Clinical Evaluation

#### 3.1.1. After 15 Days

**Figure 3:**
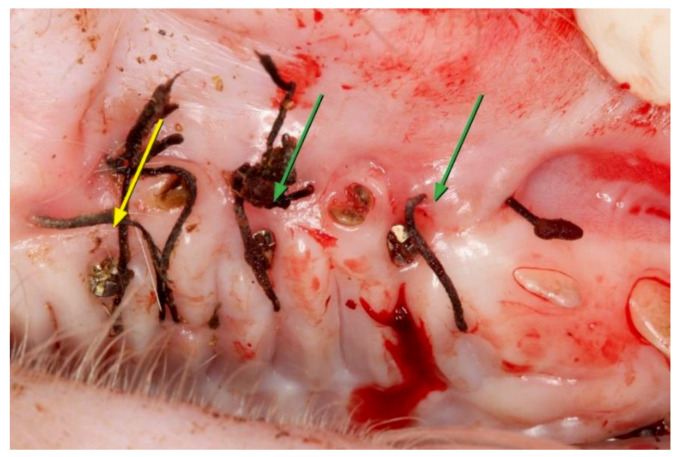
Graft condition 15 days after surgical placement. The yellow arrows indicate the location of autologous connective tissue graft (ACTG), and the green arrows indicate dermal matrix (MD).

The areas treated with MD had an apparent re-epithelialization, with a redder color than normal tissue and some whitish area. Where the micro-screw was placed, the tissue showed a purplish color, with signs of inflammation and edema, a possible result of the micro-screw’s implantation or the accumulation of bacterial plaque on the head of the screw. Surrounding the silk contour of the stitches, slight edema of the tissue was observed. The areas treated with ACTG presented a completely epithelialized surface with a slightly more reddish color compared to the healthy tissue of the animal (Figure 3).

#### 3.1.2. After 45 Days

**Figure 4:**
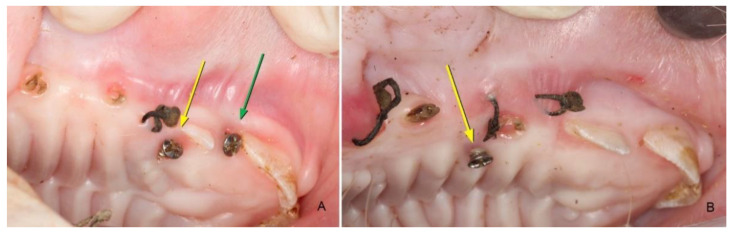
(**A**,**B**). Graft condition 45 days after surgical placement. The yellow arrows indicate the location of ACTG, and the green arrows indicate MD. The grafted areas presented a clinical healed appearance in comparison with adjacent tissues, only the tissue surrounding the screws presented a slightly edematous aspect and higher accumulation of plaque.

In the areas where the MD was grafted, clinical healing appeared complete and without differences in texture, color or appearance concerning the adjacent tissues. Keratinization was evident, and the graft surface was paler when compared to its healed state after 15 days, except in the area surrounding the micro-screw, where a higher accumulation of bacterial plaque was found and the tissue presented with a slightly edematous aspect and a color between reddish and violet. The ACTG presented a normal appearance concerning the adjacent tissues, with the same clinical appearance and color. The surface was keratinized, and the external evaluator could not distinguish this area, unaware of the treatments performed (Figure 4).

#### 3.1.3. After 90 Days

**Figure 5:**
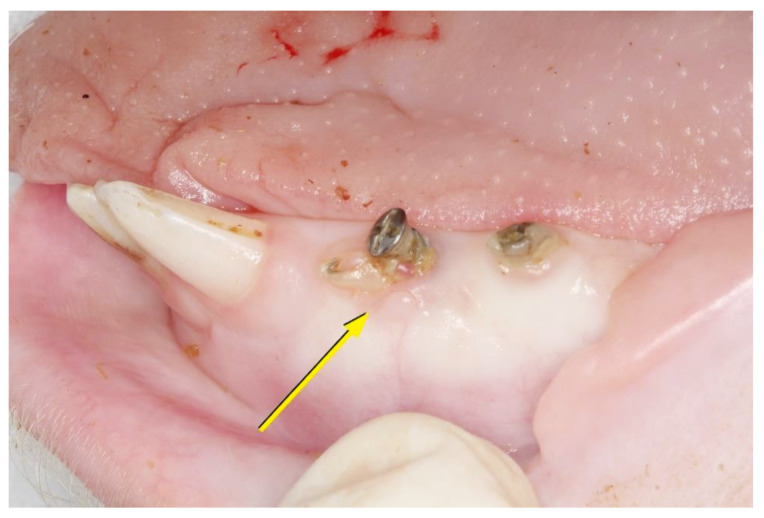
Graft condition 90 days after surgical placement. The yellow arrows indicate the location of ACTG.

The areas treated with MD had a texture and color that were not clinically distinguishable from the adjacent tissues. The area in direct contact with the implanted micro-screw presented with a different appearance and color that may be due to an inflammatory reaction to the presence of plaque. The areas treated with the ACTG had a healthy aspect, presenting the same clinical characteristics observed after 45 days (Figure 5).

The texture, color, and contour of the tissue of the areas grafted with acellular MD were in harmony with the adjacent native tissues. There was no apparent or subtle line of demarcation that would indicate the exact location and extent of the grafted material in the area. These same aesthetic results were obtained in the areas treated with the ACTG.

### 3.2. Histological Evaluation

#### 3.2.1. After 15 Days

**Figure 6:**
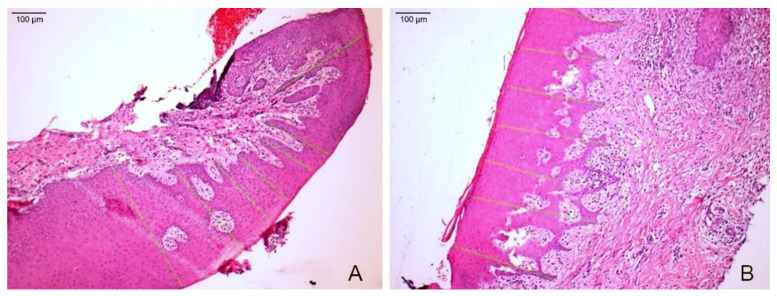
(**A**) Microphotography of the sample treated with an acellular dermal matrix of porcine origin (100×). (**B**) Microphotography of the sample treated with subepithelial autogenous connective tissue (100×). The distance from the keratin layer to the underlying connective tissue is indicated with green lines.

In the group treated with the dermal membrane, the matrix was identified on the connective tissue. The first cellular layers of immature epithelium were established, and keratinization and epithelial hyperplasia were observed. In the biopsies treated with the ACTG, a keratinized squamous epithelium, highly interdigitated with the underlying connective tissue, was observed (Figure 6).

#### 3.2.2. After 45 Days

**Figure 7:**
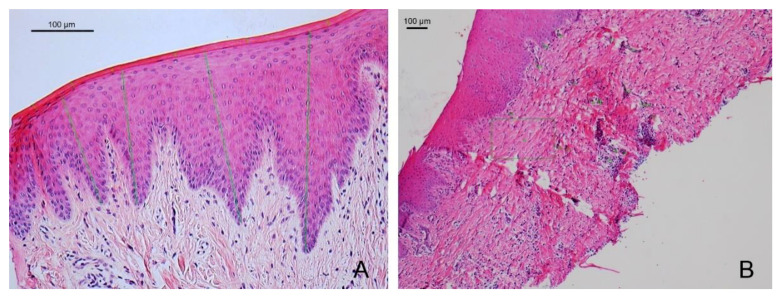
(**A**). Microphotography of the sample treated with an acellular dermal matrix at 45 days (200×). (**B**). Microphotography of the sample treated with subepithelial autogenous connective tissue at 45 days (50×). The distance from the keratin layer to the underlying connective tissue is indicated with green lines.

The biopsies corresponding to the dermal membrane showed complete epithelial healing with the establishment of a keratinized stratified epithelium. In some samples in the replacement region, the remains of the original matrices can be seen more clearly due to the degradation process. Infiltration associated with blood vessels can be seen. For the ACTG-treated group, a keratinized squamous stratified epithelium was observed, with normal perivascular infiltration and slight infiltration of deep connective tissue (Figure 7).

#### 3.2.3. After 90 Days

**Figure 8:**
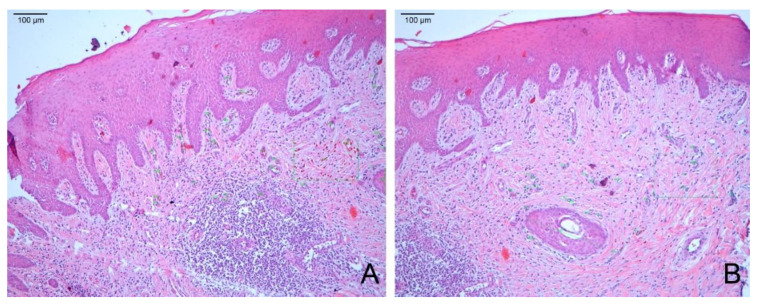
(**A**) Microphotography of the sample treated with an acellular dermal matrix at 90 days (100×). (**B**) Microphotography of the sample treated with subepithelial autogenous connective tissue at 90 days (100×).

In the biopsies treated with the dermal membrane (MD90), all locations showed completed healing, with signs of matured keratinized squamous stratified epithelial tissue, perhaps slightly thickened, over which a small band of dense orthokeratin was observed. The underlying connective tissue compartment showed correctly oriented, thick collagen fibers and normal vascularization with typical perivascular infiltration. In some of the observed samples, different findings corresponded to the detection of remains of the original unabsorbed acellular dermal matrix. In the case of the samples treated with ACTG, the keratinized squamous stratified epithelium was observed, and in the underlying connective tissue, typical mild perivascular infiltration was observed. In 30% of the samples, a high degree of inflammatory infiltration was observed in the inner area of the connective tissue, which extended towards the basal layer through the rest of the tissue, with areas of necrosis associated with this inflammatory infiltration and some areas of necrosis and acanthosis presented in the epithelium. Particular findings were observed: epithelial atrophy, short epithelial ridges, necrosis in approximately 10–20% of the connective tissue, and inflammatory infiltration in, depending on the sample, approximately 5–10% (Figure 8).

### 3.3. Keratin Layer and Epithelial Tissue Thickness

**Figure 9:**
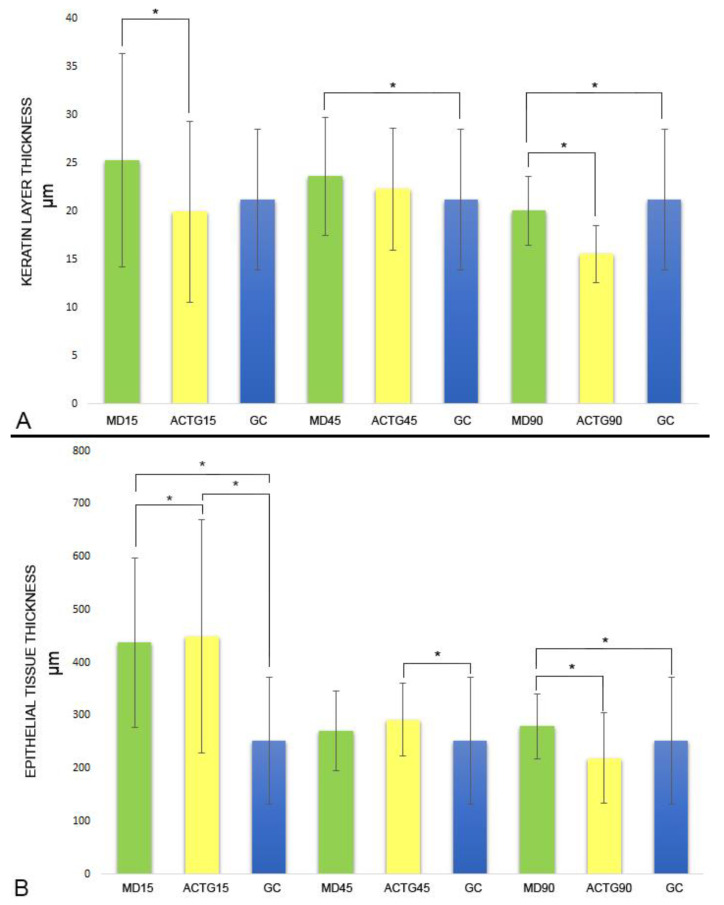
(**A**) Changes in the values of keratin layer thickness from 15 to 90 days. (**B**) Changes in the values of epithelial thickness from 15 to 90 days. * Significant differences were found when performing the Mann–Whitney U test (*p* < 0.001) (α = 0.05). MD15: MD values after 15 days; ACTG15: ACTG values after 15 days; MD45: MD values after 45 days; ACTG45: ACTG values after 45 days; MD90: MD values after 90 days; ACTG90: ACTG values after 90 days.

In all three time periods, despite a gradual decrease, the group treated with MD showed average keratin layer thickness values above that in the ACTG group (Figure 8; Supplementary Table S1). In relation to the thickness values of the epithelial tissue, epithelial hyperplasia was observed after 15 days in both the MD and the ACTG groups with respect to the CG. This tendency is maintained in the MD group, which obtained values higher than those in the CG and ACTG groups after 90 days (Figure 9; Supplementary Table S2).

## 4. Discussion

Gingival augmentation procedures have demonstrated a beneficial role in facilitating plaque control; improving patient comfort; increasing the area of attached gingiva associated with restorative, orthodontic or prosthetic dentistry; and in helping to prevent gingival recession over time [23,28,29,30]. It is estimated that 2 mm of keratinized tissue with one millimeter of tissue inserted around the teeth along with good plaque control can prevent insertion loss and gingival recession [23,28]. When keratinized gingiva gain is necessary, acellular dermal collagen matrices seem to be a good option to consider, although studies are limited [25]. For that reason, the present study evaluated the thickness of the keratin layer and the thickness of the epithelial tissue obtained after the grafting of acellular matrices as well.

When analyzing the samples histologically, at 15 days the keratin layer thickness of the samples treated with MD (25.27 μm) was significantly greater than the ACTG and CG groups, and the thickness of the MD epithelial tissue was almost double that of the CG group (437.50 ± 159.4 μm vs. 251.60 ± 120.40 μm) (*p* < 0.001). These data are consistent with that obtained by Rusu et al. [31] who observed an increase in keratinized tissue after the placement of a matrix of collagen of porcine origin. Concerning the keratinized epithelial layer, they mention that the only difference between groups at this stage was at the epithelial level, while in the newly formed gingiva, this layer was absent but presented a thicker epithelium. In the group treated with MD, unstructured epithelium with high infiltration of the underlying connective tissue was observed, while in the ACTG group, normal keratinized squamous epithelium was observed, highly interdigitated with the underlying connective tissue. In this study, a high dispersion was presented in the ACTG group concerning epithelial tissue thickness levels. This variance, however, could be due to the harvesting methodology and variances in the donor site thickness compared to the surgical beds.

After 45 days, the keratin thickness values of MD and ACTG decreased but remained higher than that of CG. In contrast, Schmitt et al. [24] compared a matrix of unidentified branded porcine collagen (CM) with ACTG at 30 days. In their results, the CM group showed greater graft contraction than the ACTG group, which significantly reduced the width of the keratinized mucosa (18.46% in the CM group and 14.59% in the ACTG group). In the present study, when measuring the thickness of the epithelial tissue, the average values of the MD group decreased considerably (270.76 ± 75.19 μm) concerning the averages obtained at 15 days; however, both the ACTG and MD groups obtained significantly higher values than the EC group. For both groups, the samples observed showed complete epithelial healing with the establishment of a keratinized stratified epithelium. In biological terms, this could explain why all of the values of this variable remained within a range of normality; however, we could not find any studies that evaluated the thickness of the epithelial tissue during this period.

At 90 days in the MD group, a keratinized squamous stratified epithelial tissue with a dense band of orthokeratin and a thickness similar to those presented in the CG was identified, although it was not statistically significant, and the ACTG decreased considerably compared to CG. These results are consistent with those obtained by Nevins et al. [32] who also observed at 90 days the presence of a keratinized epithelium with a small dense band of orthokeratinization in all samples at the sites treated with an extracellular matrix derived from porcine small intestinal submucosa. Similarly, the histological results obtained by Schmitt et al. [24], both for the porcine collagen matrix and the ACTG-treated group, showed a multilayered keratinized squamous epithelium structure with similar keratin expression patterns in both groups. 

Concerning epithelial thickness, at 90 days, the MD group was larger (281.49 ± 63.67 μm) and showed statistically significant differences compared to the CG group (251.60 ± 10.6 μm) (*p* < 0.001). A significant increase was also observed in the MD group when compared to the values obtained at 45 days (270.76 ± 7.11 μm) (*p* = 0.023). In contrast, the ACTG decreased in size compared to the measures obtained at 45 days (292.02 ± 7.13 μm < 219.71 ± 5.98 μm) until lower values were obtained for the CG group. Matoh et al. [33] also identified an increase in thickness after 6 and 12 months in both acellular dermal matrix-treated and autogenous connective tissue graft-treated areas. However, the final thickness was significantly higher in the sites treated with autogenous connective tissue graft as a control group (0.9 ± 0.2 mm < 2.1 ± 0.2 mm) (*p* < 0.01) compared to those treated with MD (0.3 ± 0.2 mm < 1.4 ± 0.3 mm). The data of the present study are also in line with those obtained by Nevins et al. [32], who observed an average epithelial thickness of 294 ± 17.9 μm after 13 weeks in 6 patients who were treated with the matrix derived from porcine small intestinal submucosa. These results for the study matrix were similar to those obtained in our study, with an average epithelial thickness of 281.49 μm on the MD90 group.

The data from our study differ from those obtained by Wei et al. [21] who compared clinically and histologically an unidentified branded acellular dermal matrix (ADM) with the ACTG by exposing the matrix in the oral cavity to gain keratinized tissue. They reported a less effective and predictable outcome in terms of matrix gain (2.59 ± 0.92 mm) compared to the ACTG (5.57 ± 0.44 mm) due to considerable graft contraction. Schmitt et al. [24] also reported a slightly greater reduction in the porcine collagen matrix (7.35%) over the ACTG group (7.11%) at 90 days, as did Harris et al. [34], who reported a keratinized gum gain after 3 months of 4.8 mm for the ACTG group and 4.7 mm for the acellular dermal matrix group [35]. In some of the samples observed, we found different findings corresponding to the detection of remains of the unabsorbed, original acellular dermal matrix. Thus, further studies over a longer duration are necessary to evaluate the biological impact of this intervention.

One major concern of this study regards the standardization of the grafts’ dimensions. Although the acellular dermal matrix always presents the same thickness, even if performed by the same expert, manual harvesting of the autologous graft can nonetheless lead to a variation in the samples used, altering thickness parameters. Within these limitations, after three months’ time in this pig model study the MD group outperformed the ACTG group in terms of keratin layer and epithelial tissue thickness. Considering the clinical morbidity and limited availability of the autologous graft techniques, the use of this xenogeneic material could be a valid alternative. Current extrapolation of this study design to a human model is not possible due to ethical considerations; therefore, further studies using different animal models could provide insights into the biologic effects of this regenerative method.

## Data Availability

The data presented in this study are available on request from the corresponding author after obtaining permission of authorized person.

## Supplementary Materials

The following are available online at https://www.mdpi.com/1996-1944/14/2/272/s1, Table S1. Keratin layer thickness in samples treated with acellular dermal matrix and subepithelial autogenous connective tissue compared to the control tissue, Table S2. Epithelial tissue thickness in samples treated with acellular dermal matrix and subepithelial autogenous connective tissue compared to the control tissue.
