# Supplementary material for: Clinical and Histological Differences between Guided Tissue Regeneration with Acellular Dermal Matrix of Porcine Origin and Autologous Connective Tissue: An Animal Study
Source: Materials (Basel). 2021 Jan 7;14(2):272. doi: 10.3390/ma14020272 (PMC7827452; doi:10.3390/ma14020272)
Supplement: Supplementary file 1 [file materials-14-00272-s001.pdf]

materials

Supplementary Material

Table 1. Keratin layer thickness in samples treated with acellular dermal matrix and subepithelial autogenous connective tissue compared to the control tissue.

Groups	*n*	Mean (μm)	SEM	SD	*p-value*		
		After 15 days			CG	MD	ACTG
**CG**	106	21.20	0.71	7.34	-	0.003*	0.010*
**MD**	123	25.28	0.996	11.04	0.003*	-	<0.001*
**ACTG**	75	19.95	1.09	9.4	0.010*	<0.001*	-
		After 45 days			CG	MD	ACTG
**CG**	106	21.20	0.71	7.34	-	<0.001*	0.016*
**MD**	124	23.61	0.55	6.13	<0.001*	-	0.599
**ACTG**	87	22.26	0.67	6.29	0.016*	0.599	-
		After 90 days			CG	MD	ACTG
**CG**	106	21.20	0.71	7.34	-	0.204	<0.001*
**MD**	290	19.46	0.25	4.29	0.204	-	<0.001*
**ACTG**	223	15.53	0.20	2.99	<0.001*	<0.001*	-

CG: Control group; MD: group treated with an acellular dermal matrix of porcine origin; ACTG: group treated with autologous connective tissue graft; *Significant differences were found when realizing the Mann-Whitney U test ($p<0.05$).

Table Supplementary 2. Epithelial tissue thickness in samples treated with acellular dermal matrix and subepithelial autogenous connective tissue compared to the control tissue.

Groups	*n*	Mean (μm)	SEM	SD	*p-value*		
		After 15 days			CG	MD	ACTG
**CG**	106	251.6	10.6	120.4	-	<0.001*	<0.001*
**MD**	198	437.5	11.3	159.4	<0.001*	-	0.847
**ACTG**	53	449.4	30.4	221	<0.001*	0.847	-
		After 45 days			CG	MD	ACTG
**CG**	106	251.60	10.60	120.40	-	0.002*	<0.001*
**MD**	112	270.76	7.11	75.19	0.002*	-	0.012*
**ACTG**	94	292.02	7.13	69.15	<0.001*	0.012*	-
		After 90 days			CG	MD	ACTG
**CG**	106	251.60	10.60	120.40	-	<0.001*	0.012*
**MD**	284	281.49	3.78	63.67	<0.001*	-	<0.001*
**ACTG**	204	219.71	5.98	85.36	0.012*	<0.001*	-

CG: Control group; MD: group treated with an acellular dermal matrix of porcine origin; ACTG: group treated with autologous connective tissue graft; *Significant differences were found when realizing the Mann-Whitney U test ($p<0.05$).
